# Infection by dsRNA viruses is associated with enhanced sporulation efficiency in *Saccharomyces cerevisiae*

**DOI:** 10.1002/ece3.8558

**Published:** 2022-01-22

**Authors:** Thomas J. Travers Cook, Christina Skirgaila, Oliver Y. Martin, Claudia C. Buser

**Affiliations:** ^1^ 27219 Institute of Integrative Biology ETH Zürich Zürich Switzerland; ^2^ 28499 Department of Aquatic Ecology Eawag Dübendorf Switzerland; ^3^ 27219 Department of Biology ETH Zürich Zürich Switzerland

**Keywords:** dsRNA viruses, host manipulation, insect vectors, mutualism–parasitism continuum, phenotypic plasticity, selfish genetic elements, sexual reproduction, yeast

## Abstract

Upon starvation diploid cells of the facultative sexual yeast *Saccharomyces cerevisiae* undergo sporulation, forming four metabolically quiescent and robust haploid spores encased in a degradable ascus. All endosymbionts, whether they provide net benefits or costs, utilize host resources; in yeast, this should induce an earlier onset of sporulation. Here, we tested whether the presence of endosymbiotic dsRNA viruses (M satellite and L‐A helper) correspond with higher sporulation rate of their host, *S*. *cerevisiae*. We find that *S*. *cerevisiae* hosting both the M and L‐A viruses (so‐called “killer yeasts”) have significantly higher sporulation efficiency than those without. We also found that the removal of the M virus did not reduce sporulation frequency, possibly because the L‐A virus still utilizes host resources with and without the M virus. Our findings indicate that either virulent resource use by endosymbionts induces sporulation, or that viruses are spread more frequently to sporulating strains. Further exploration is required to distinguish cause from effect.

## Graphical abstract

Sporulation in yeast is induced by starvation; resource exploitation by endosymbiont should induce earlier sporulation. We find that the presence of two dsRNA viruses is linked to enhanced sporulation in *Saccharomyces cerevisiae*. However, further research is required to determine whether high sporulation leads to infection or infection leads to higher sporulation.

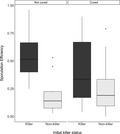

## 1 INTRODUCTION

Sporulation is a remarkable instance of phenotypic plasticity. It enables organisms to adapt to adverse nutrient‐depleted conditions and survive in a metabolically quiescent and stress resistant state until vegetative growth is once again permitted (Driks, 2002; Knight & Goddard, 2016). Whilst diploid cells of the ascomycete yeast *Saccharomyces cerevisiae* primarily replicate asexually via mitosis, upon inhospitable conditions, they can undergo meiosis to form a tetrad of four haploid spores of two distinct mating types (a and α) (Honigberg & Purnapatre, 2003; Neiman, 2005; Piccirillo & Honigberg, 2010). Sporulation is a quantitative trait exhibiting complex mapping from genotype to phenotype (Ben‐Ari et al., 2006; Deutschbauer & Davis, 2005; Gerke et al., 2009); a number of candidate genes have been detected with nonsynonymous mutations associated with variation in sporulation (Tomar et al., 2013).

It is widely recognized that sporulation enables dispersal by insect vectors. Drosophilids are closely associated with yeast throughout their life history, and play a central role in their dispersal (Dobzhansky et al., 1956; Gilbert, 1980; Reuter et al., 2007; Starmer & Fogleman, 1986). Whilst the acidic conditions of insect digestive systems destroy vegetative cells, spores generally survive passage (Coluccio et al., 2008; Reuter et al., 2007). Sporulation is primarily a starvation response, as the absence of nitrogen and fermentable carbon initiate the developmental pathway of meiosis and sporulation (Freese et al., 1982; Mata et al., 2002; Neiman, 2011; Primig et al., 2000). However, spores encounter digestive enzymes and altered pH in the digestive tract, and they generally remain unscathed. This would suggest that predation by insects has influenced their evolution (Thomasson et al., 2021).

Whilst spores survive due to the protective chitosan and dityrosine layers of the spore wall, the ascus holding sister spores together is broken down in the digestive tract (Coluccio et al., 2008). This provides potential for outcrossing between spores from different asci upon the return of resource‐rich conditions (Reuter et al., 2007). For *S*. *cerevisiae*, this is typically achieved by dispersal to a new fruit resource. Sporulation, and subsequent dispersal by insect vectors, thus provides a method by which *S*. *cerevisiae* can increase genetic diversity (Stefanini et al., 2016); this is particularly important for an asexual homothallic species with a tendency for automixis (Herskowitz & Jensen, 1991; Magwene et al., 2011; Zeyl & Bell, 1997). Increasing genetic diversity is useful prior to dispersal as it increases the likelihood that one or more daughter cells will possess a fitness sufficient to persist in the novel environment to be encountered (Otto & Lenormand, 2002).

Sexual reproduction is risky, however, because it enables the spread of parasitic genetic elements. It is believed that RNA interference (RNAi) and programmed cell death have evolved to attenuate infective elements (Drinnenberg et al., 2009; Gao, Chau, & Meneghini, 2019). For example, dsRNA M satellite and L‐A helper viruses are absent from yeast reconstituted with an RNAi pathway, which is naturally absent (Drinnenberg et al., 2011). Moreover, Gao, Chau, Chowdhury, et al. (2019) found the same viruses were attenuated during sporulation by the *NUC1* nuclease.

Biotic influences on sporulation efficiency, defined as the proportion of cells in a culture to sporulate, have been overlooked. Whether providing a net benefit or cost to the host, the L‐A virus (and indirectly the M virus) utilizes host resources. As starvation induces sporulation, the presence of resource‐utilizing intracellular endosymbionts should enhance the onset of starvation and sporulation under resource‐limiting environments. This should be a natural by‐product of their infectivity and virulence. We refer to this as the “infection by‐product hypothesis,” whereby enhanced sporulation is an unavoidable by‐product of infection, with its onset determined by parasite virulence and environmental resource availability. As starvation is the trigger of the sporulation pathway, we expect endosymbiotic infections to enhance sporulation no matter the strain explored. On the contrary, endosymbiont‐free yeasts should have lower sporulation efficiencies, which may be detrimental and non‐adaptive in competitive environments.

To investigate whether endosymbiosis is linked to enhanced sporulation we made use of the *S*. *cerevisiae* killer yeast system, which is composed of the yeast host, and two dsRNA viruses (M satellite and L‐A helper viruses). The combination of M and L‐A viruses encodes an antimycotic toxin for use by their host in interference competition, known collectively as the killer phenotype (Boynton, 2019; Wickner, 1996; Zhu et al., 1993). The dsRNA L‐A genome encodes replication and transcription of the M virus and itself. The M genome is hosted and propagated by the L‐A virion on which it is completely dependent. The M virus encodes toxin production and immunity to said toxin. These viruses provide context‐dependent benefits to their hosts, depending on the level of competition present. We hypothesize yeast exhibiting the killer phenotype will have significantly enhanced sporulation efficiency relative to non‐killers. We also predict that removing the M virus from killers will not significantly lower sporulation efficiency because the L‐A virus will continue to utilize the hosts resources in the absence of the M virus.

## 2 MATERIALS AND METHODS

### 2.1 Study system

In total, 11 *S*. *cerevisiae* strains – five non‐killers and six killers – were used (see Table 1). Of the killer strains, three belonged to the K1 killer‐type, two to K2 and one to Klus. For each killer strain sample, a corresponding M virus‐free strain was created from treatment with elevated temperature (Wickner, 1974), anisomycin (Fredericks et al., 2021) or cycloheximide (Fink & Styles, 1972) to destroy genetic material of the M virus. This procedure is commonly referred to as “curing” the strains. Killer strains and their cured equivalent were donated and cured with the aforementioned methods by the groups of Paul Rowley and Nieves Rodríguez‐Cousiño (Fredericks et al., 2021; Rodriguez‐Cousino et al., 2013). For a control, all non‐killer strains were additionally independently exposed to elevated temperature (a widely utilized curing method) prior to the experiment (Wickner, 1974), as this would allow us to establish whether curing of the killer factor per se or simply a curing process can influence sporulation efficiency. This also leads to a full factorial design of cured non‐killers, non‐killers, cured‐killers and killers. Background information on each strain can be found in the respective collections of each strain (see Table 1).

**TABLE 1:** Strains of *Saccharomyces cerevisiae* used and their killer phenotypes

Strains	Killer	Totivirus	Satellite	Toxin	Collection
DBVPG1895	No	No	No	No	Liti 1011 (Peter et al., 2018)^a^
DBVPG6302	No	No	No	No	Liti 1011 (Peter et al., 2018)^a^
DBVPG4410	No	No	No	No	Liti 1011 (Peter et al., 2018)^a^
DBVPG4460	No	No	No	No	Liti 1011 (Peter et al., 2018)^a^
DBVPG6223	No	No	No	No	Liti 1011 (Peter et al., 2018)^a^
NCYC 190	Killer	Yes	Yes	K1	NCYC (Fredericks et al., 2021)^a^, ^b^
Y−2429	Killer	Yes	Yes	K1	NRRL (Fredericks et al., 2021)^a^, ^c^
YJM1307	Killer	Yes	Yes	K1	FGSC (Fredericks et al., 2021)^a^, ^d^
NCYC1001	Killer	Yes	Yes	K2	NCYC (Fredericks et al., 2021)^a^, ^b^
Ca7	Killer	Yes	Yes	K2	Cádiz, Spain (Rodriguez‐Cousino et al., 2013)^e^
Ex198	Killer	Yes	Yes	Klus	Ribera del Guadiana, Spain (Rodriguez‐Cousino et al., 2013)^e^

^a^ Donated by Paul Rowley.

^b^ National Collection of Yeast Cultures.

^c^ Agricultural Research Service Culture Collection (Northern Regional Research Laboratory) Database.

^d^ Fungal Genetics Stock Center.

^e^ Donated by Nieves Rodríguez‐Cousiño.

### 2.2 Sporulation

Three independent replicates per *S*. *cerevisiae* strain were cultivated on standard YPD media (1% yeast extract, 2% peptone, and 10% glucose) at 25°C overnight, and subsequently the optical densities (OD) of the growing yeast cells were measured (SpectraMax 19, software: SoftMax Pro 6.2.2). When the OD was between 0.6 and 0.8, 50 ml of yeast were washed according to the protocol of Knight and Goddard (2016), with the following modifications: washed twice with 3 ml of sterile water. 0.5 ml of each yeast strain was plated on sporulation plates (40 mm plates, sporulation media: 1% potassium acetate, 0.1% yeast extract, 0.05% glucose, 2% agar, 25 mg/L zinc sulfate). Yeast plates were inoculated for 7 days at 25° C. Finally, we evaluated 100 cells for each strain replicate under the microscope and classified their sporulation status to calculate the sporulation efficiency, defined as the proportion of cells in a culture that have sporulated (Knight & Goddard, 2016).

### 2.3 Statistical analysis

All analyses were performed in R version 4.0.1 (R Development Core Team, 2020). To establish whether the presence of viruses or heat stress affects the proportion of sporulating cells, a linear mixed effect model was constructed in the package “lme4” (Bates et al., 2015), with strain as a random effect. We used the proportion of sporulating cells as the response variable. An interaction term of previous killer phenotype or previous killer type, and exposure to a curing treatment was created, leading to a full factorial design. Using simultaneous inference in general parametric methods (contrast analysis) in the package multcomp (Horthorn et al., 2008), any significant differences in sporulation rate between factors was established. Bonferroni corrections were applied. We treated strain as a random variable throughout to establish any effects on sporulation efficiency of the killer phenotype after accounting for variation in strain. The percentage of total variance explained by the strain was also calculated.

## 3 RESULTS

**FIGURE 1:**
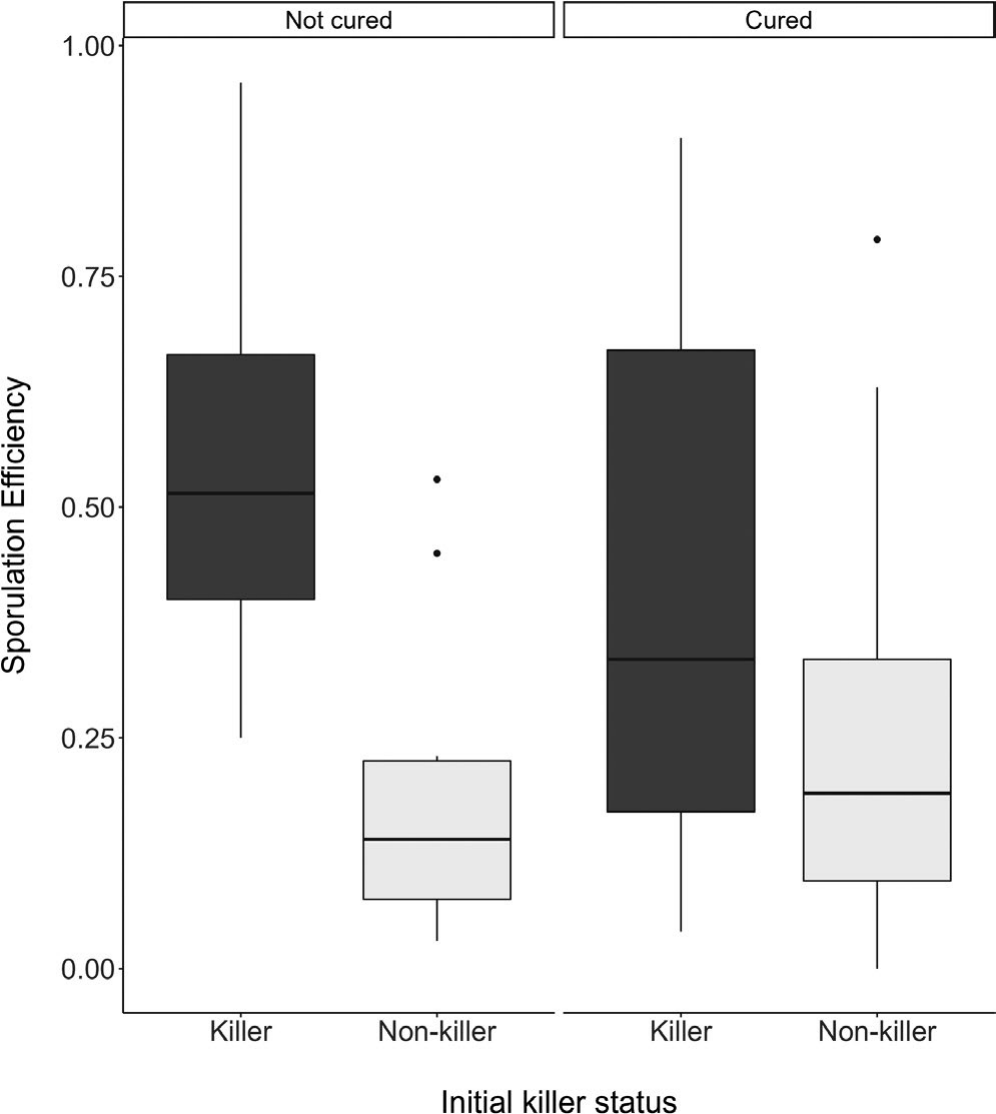
The proportion of cells to sporulate is plotted against the killer phenotype status (killer and non‐killer). The original killer phenotype status is faceted according to whether strains had been cured of the viruses or not. The box represents the interquartile range, the line extremities represent the range, the horizontal line in each box represents the median and points indicate outliers

The proportion of cells to sporulate was consistently higher for killers than for non‐killers (see Table 2, Killers vs. L‐A only and initial non‐killers, and killers vs. non‐cured non‐killers) (Figure 1). In general, curing did not have a significant effect on sporulation (see Table 2, non‐cured vs. cured, killer vs. L‐A only, non‐cured non‐killer vs. cured non‐killer), though non‐cured killers tended to have higher sporulation rates than their cured equivalents (*F* = 3.500, *p* = .0669; linear mixed effect model; Figure 1). This indicates that killer yeasts have enhanced sporulation rates, but that the curing methods used (which remove the M, but not the L‐A) are not capable of lowering this rate significantly. Strain was found to only explain 10.39% of variance in sporulation efficiency after the fixed effects.

**TABLE 2:** Contrast analysis showing the effect of killer phenotype and removal of viruses on sporulation. Here, the term “killers” refers to all strains that have the killer phenotype. “L‐A only” refers to strains that were originally killers, but have since been cured of the M virus. “Initial non‐killers” consists of both non‐cured and cured non‐killers

Comparison	Estimate	*SE*	*Z* value	*p*
Killers vs. L‐A only and initial non‐killers	0.26644	0.06873	3.877	.000953***
Killers and L‐A only *vs*. initial non‐killers	0.25856	0.07162	3.610	.002754**
Killers vs. non‐cured non‐killers	0.18072	0.04515	4.003	.000563***
Killers vs. L‐A only	0.07056	0.03708	1.903	.513516
L‐A only vs. non‐cured non‐killers	0.11017	0.04515	2.440	.132167
L‐A only vs. initial non‐killers	0.132167	0.08065	2.331	.177723
Non‐cured vs. cured	0.03822	0.05500	0.695	1.000000
Non‐cured non‐killer vs. cured non‐killer	−0.03233	0.04062	−0.796	1.000000

**FIGURE 2:**
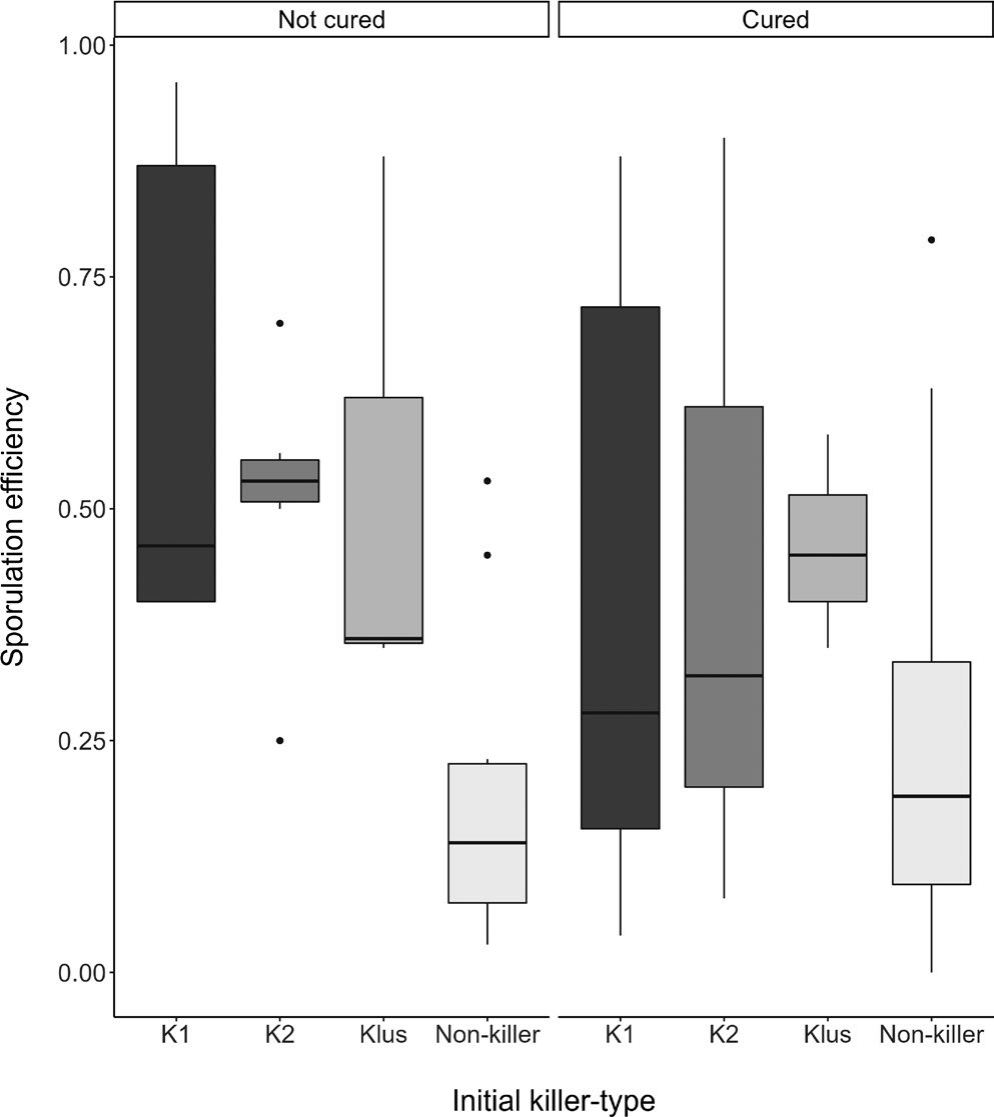
The proportion of cells to sporulate is plotted against the initial killer‐type (K1, K2, Klus, and non‐killer). The original killer‐type status is faceted for whether they have been cured of the viruses. The box represents the interquartile range, the line extremities represent the range, the horizontal line in each box represents the median and points acknowledge outliers

Killer type did not affect sporulation rate, but in general the different killer types had higher sporulation efficiencies compared to non‐killers (see Figure 2, non‐cured non‐killers vs. non‐cured K1, K2, and Klus: Estimate: −0.352078, *SE*: 0.101233, *Z*‐value: −3.478, *p* = .0091). Furthermore, non‐cured K1 was the only killer found to be significantly different from non‐killers (non‐cured non‐killers vs. non‐cured K1: Estimate: −0.195765, *SE*: 0.057697, *Z*‐value: −3.393, *p* = .0124).

**FIGURE 3:**
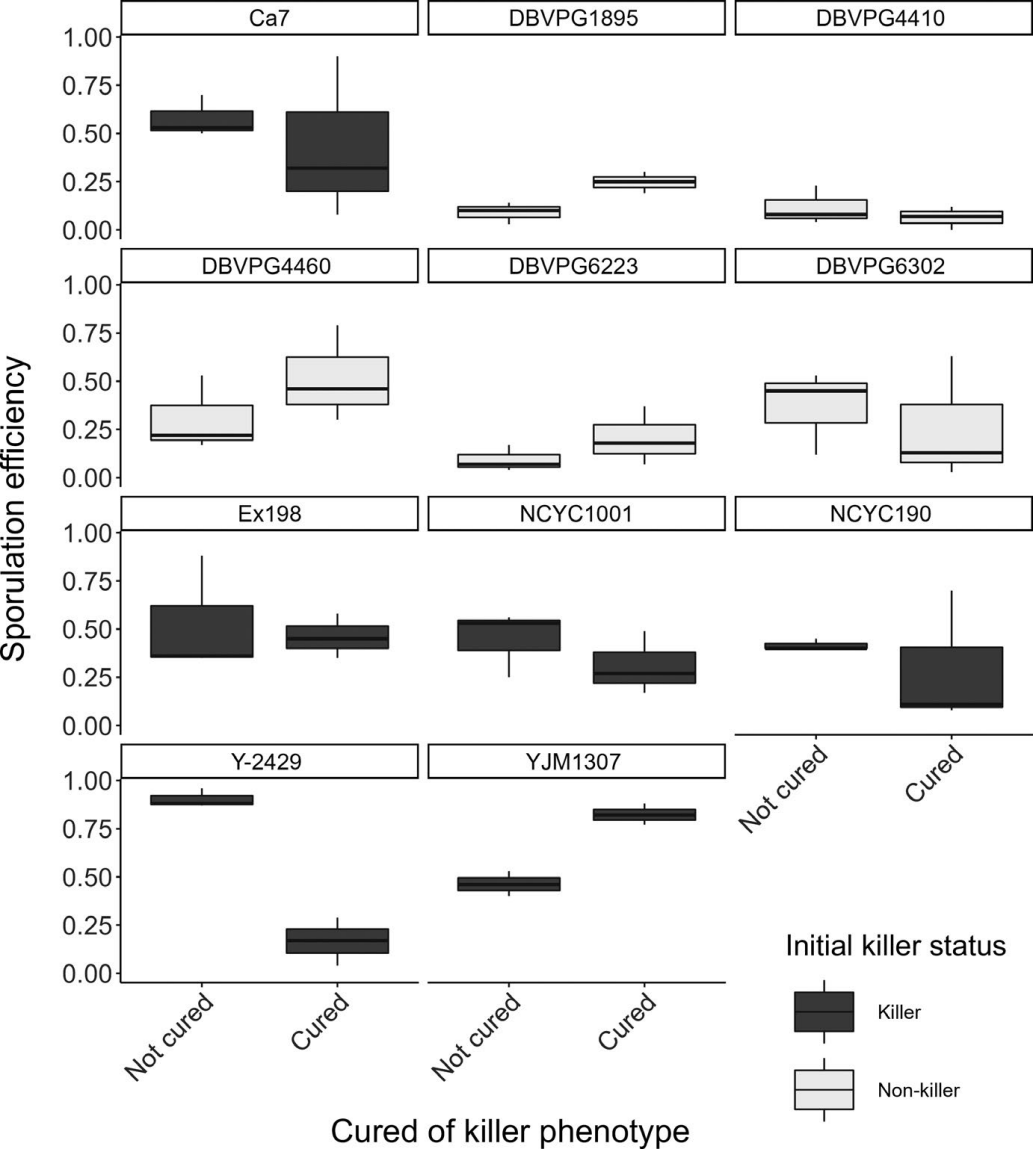
The variation in sporulation is demonstrated across the cured and uncured equivalents of all strains used. Boxplots are color coded by whether they were initially a killer (killers = dark grey, non‐killers = light grey), and are faceted by whether curing was applied to them. The box represents the interquartile range, the line extremities represent the range, and the horizontal line in each box represents the median

Finally, we find significant variation in the propensity to sporulate across all the strains used (see Figure 3). For non‐killer strains, there appears to be no general pattern concerning whether curing increases or decreases sporulation efficiency. All killer strains except YJM 1307 generally had higher sporulation efficiencies prior to curing.

## 4 DISCUSSION

The dsRNA M satellite and L‐A helper viruses in *S*. *cerevisiae* are known for encoding toxin production as an interference competition mechanism. Recently, Buser et al. (2021) found that yeast hosting dsRNA viruses were more attractive to *Drosophila simulans*, a Drosophilid responsible for yeast dispersal (Buser et al., 2014). Surviving dispersal via gut passage requires sporulation. Here, we find evidence that the killer phenotype (the presence of the M satellite and L‐A helper viruses) is significantly associated with higher rates of sporulation. This finding hints at an acutely intertwined relationship between the sexual life‐stage of *S*. *cerevisiae* and infection by viruses. It suggests that the dsRNA viruses play roles in *S*. *cerevisiae* life history and evolutionary ecology beyond interference competition, despite the potential survival ramifications for the viruses (Gao, Chau, Chowdhury, et al., 2019). In particular, it could mean that viruses play a role in their host's expansion into highly competitive fermentation sites by enhancing sporulation rates, as is theorized of endosymbionts (Joy, 2013; Sudakaran et al., 2017). Nonetheless, we merely present a positive statistical association between the presence of dsRNA viruses and the sporulation efficiency, with the cause as of yet unknown. Here, we outline three possible explanations for this observation.

Our finding that non‐cured killers have significantly higher sporulation rates than the non‐killers supports our infection by‐product hypothesis, that endosymbiont infectivity and virulence enhance sporulation efficiency (see Table 2). Starvation halts cell growth by mitotic division, inducing in its place the meiotic regulatory pathway (Honigberg & Purnapatre, 2003). Nitrogen and fermentable carbon limitations are primarily responsible for inciting sporulation, though limitations of phosphate, sulfate, guanine, and methionine are also demonstrated causes (Freese et al., 1982). The presence of endosymbiotic viruses may cause resource limitation, as resource usage is part of any infection. L‐A helper and M satellite viruses make use of free‐floating nucleotides (Marquina et al., 2002; Schmitt & Breinig, 2006). If dsRNA viruses are diverting resources away from their host, meiotic gene expression may be triggered by a limitation of guanine or another nucleotide.

We found no significant difference in sporulation rate between current and former killer yeast, though there does appear to be a slight general decline in sporulation after curing (see Figure 1). The curing techniques used (elevated temperature, anisomycin, or cycloheximide application) remove the M satellite virus, but the L‐A virus typically persists. The M virus (often termed a virophage) is housed and propagated in the host cell by the L‐A virus (Schmitt & Breinig, 2006). The L‐A virus should harness a set quantity of resources from the host independent of the presence of the M virus; however, if the requirement to share resources with the M virus is absent, the LA viral copy number should be higher. One killer (YJM1307) had higher sporulation efficiency after curing which could be due to curing‐induced modifications to sporulation genes (Lorenz & Cohen, 2014), which may also apply to some non‐killers (see Figure 3). The high variation in the sporulation efficiency of yeast carrying a virus could be determined by viral load, which plausibly influences host metabolic costs of endosymbiosis. Though not explored in this paper, the degree of coadaptation likely affects whether hosts have evolved stabilizing mechanisms, such as sanctions of viral load (Gao, Chau, Chowdhury, et al., 2019; Wickner & Edskes, 2015), in order to compensate for the metabolic costs of endosymbiosis.

Alternatively, enhanced sporulation in killer yeast may be an adaptation to predation. Thomasson et al. (2021) demonstrated that sporulation is an adaptive trait, and that insect passaging selects for yeasts with higher rates of spore production. *Drosophila* attraction varies across yeast strains (Buser et al., 2014). Strains that are more attractive would therefore have higher sporulation rates as insect vectors disperse them more frequently. It was recently shown that killer yeasts are generally more attractive than non‐killers (Buser et al., 2021). An alternative explanation for the higher sporulation rate in killers could therefore be that it is an adaptive trait making up for their higher attraction to insect vectors. A proximate explanation for their higher attraction may be a change in their volatile composition resulting from virus infection, which remains untested. An ultimate explanation could be that killer yeasts are found more frequently in highly competitive fermentation environments meaning fruit flies consume them more regularly.

A third explanation is that viruses do not induce higher rates of sporulation. There is standing variation in the propensity of hosts to sporulate, demonstrated here (see Figure 3). This is likely due to variation in their abilities to acquire resources from the external environment and subsequent variation in the timing of starvation. Candidate genes have been linked to variation in sporulation efficiency (Tomar et al., 2013), whereby the significance of the nonsynonymous mutation is determined by the stage of sporulation at which the gene is expressed (Lorenz & Cohen, 2014). As these strains sporulate more frequently, they are also more likely to undergo sexual reproduction, and outcross with non‐clonal spores. If sexual reproduction is the only means of transmitting dsRNA viruses beyond the host lineage, viruses are only transmitted to strains that are sporulating. Therefore, strains with higher sporulation rates are more likely to be infected by viruses and other genetic elements. However, the cytosolic nucleases produced during meiosis degrade the M and L‐A viruses of *S*. *cerevisiae* (Gao, Chau, Chowdhury, et al., 2019); it seems counterintuitive for frequently sporulating strains to host viruses when immune responses in sporulation actively degrade any foreign genetic elements. It appears that *NUC1* does act as a host stabilizing mechanism to prevent uncontrolled proliferation of their cytosolic mycoviruses. However, it remains to be seen whether these dsRNA viruses can persist in their host if their pre‐sporulation viral load is above a certain threshold. If so, this explanation is quite probable; however, if not possible, it gives credence to our infection by‐product hypothesis, that sporulation is an unavoidable consequence of infection.

## 5 CONCLUSION

Among the detrimental consequences of sexual reproduction is the transmission of parasitic genetic elements, of which resource exploitation is a consequence. Here, we provide evidence linking dsRNA viruses to enhanced sporulation in *S*. *cerevisiae*. Our findings have three possible explanations: (1) high sporulation rates are a consequence of the virulence of infectious virions; (2) strains with higher sporulation rates outcross more frequently, thus providing an opportunity for infection by parasitic genetic elements; (3) sporulation is an adaptive trait of killer yeast to account for their higher attractiveness to dispersal vectors. Our findings hint at a deeply interwoven relationship between sexual reproduction and parasitism in *S*. *cerevisiae*. Future work is required to establish causation.

## CONFLICT OF INTEREST

The authors declare no conflict of interest. The funders had no role in the design of the study; in the collection, analyses, or interpretation of data; in the writing of the manuscript, or in the decision to publish the results.

## AUTHOR CONTRIBUTIONS

**Thomas J. Travers Cook:** Conceptualization (equal); Data curation (equal); Formal analysis (equal); Investigation (equal); Methodology (equal); Validation (equal); Visualization (lead); Writing – original draft (lead); Writing – review & editing (lead). **Christina Skirgaila:** Conceptualization (equal); Data curation (equal); Formal analysis (supporting); Methodology (equal); Writing – original draft (supporting). **Oliver Y. Martin:** Conceptualization (equal); Supervision (equal); Validation (equal); Writing – review & editing (equal). **Claudia C. Buser:** Conceptualization (lead); Data curation (supporting); Formal analysis (equal); Funding acquisition (lead); Investigation (equal); Methodology (equal); Project administration (lead); Resources (lead); Supervision (lead); Validation (equal); Writing – original draft (supporting); Writing – review & editing (equal).

## Data Availability

The Data used in the analysis of this paper can be found in the Dryad Data Repository (https://doi.org/10.5061/dryad.x0k6djhm8).

## References

[ece38558-bib-0001] Bates, D. , Mächler, M. , Bolker, B. , & Walker, S. (2015). Fitting linear mixed‐effects models using lme4. Journal of Statistical Software, 67, 1–48. 10.18637/jss.v067.i01

[ece38558-bib-0002] Ben‐Ari, G. , Zenvirth, D. , Sherman, A. , David, L. , Klutstein, M. , Lavi, U. , Hillel, J. , & Simchen, G. (2006). Four linked genes participate in controlling sporulation efficiency in budding yeast. PLoS Genetics, 2, e195. 10.1371/journal.pgen.0020195 17112318 PMC1636695

[ece38558-bib-0003] Boynton, P. J. (2019). The ecology of killer yeasts: Interference competition in natural habitats. Yeast, 36, 473–485. 10.1002/yea.3398 31050852

[ece38558-bib-0004] Buser, C. C. , Jokela, J. , & Martin, O. Y. (2021). Scent of a killer: How could killer yeast boost its dispersal? Ecology and Evolution, 11, 5809–5814. 10.1002/ece3.7534 34141185 PMC8207343

[ece38558-bib-0005] Buser, C. C. , Newcomb, R. D. , Gaskett, A. C. , & Goddard, M. R. (2014). Niche construction initiates the evolution of mutualistic interactions. Ecology Letters, 17, 1257–1264. 10.1111/ele.12331 25041133

[ece38558-bib-0006] Coluccio, A. E. , Rodriguez, R. K. , Kernan, M. J. , & Neiman, A. M. (2008). The yeast spore wall enables spores to survive passage through the digestive tract of *Drosophila* . PLoS One, 3, e2873. 10.1371/journal.pone.0002873 18682732 PMC2478712

[ece38558-bib-0007] Deutschbauer, A. M. , & Davis, R. W. (2005). Quantitative trait loci mapped to single‐nucleotide resolution in yeast. Nature Genetics, 37, 1333–1340. 10.1038/ng1674 16273108

[ece38558-bib-0008] Dobzhansky, T. , Cooper, D. M. , Phaff, H. J. , Knapp, E. P. , & Carson, H. L. (1956). Studies on the ecology of *Drosophila* in the Yosemite Region of California. 4. Differential attraction of species of *Drosophila* to different species of yeasts. Ecology, 37, 544–550. 10.2307/1930178

[ece38558-bib-0009] Driks, A. (2002). Overview: Development in bacteria: Spore formation in *Bacillus subtilis* . Cellular and Molecular Life Sciences, 59, 389–391. 10.1007/s00018-002-8430-x 11964116 PMC11337451

[ece38558-bib-0010] Drinnenberg, I. A. , Fink, G. R. , & Bartel, D. P. (2011). Compatibility with killer explains the rise of RNAi‐deficient fungi. Science, 333, 1592. 10.1126/science.1209575 21921191 PMC3790311

[ece38558-bib-0011] Drinnenberg, I. A. , Weinberg, D. E. , Xie, K. T. , Mower, J. P. , Wolfe, K. H. , Fink, G. R. , & Bartel, D. P. (2009). RNAi in budding yeast. Science, 326, 544–550. 10.1126/science.1176945 19745116 PMC3786161

[ece38558-bib-0012] Fink, G. R. , & Styles, C. A. (1972). Curing of a killer factor in *Saccharomyces cerevisiae* . Proceedings of the National Academy of Sciences of the United States of America, 69, 2846–2849. 10.1073/pnas.69.10.2846 4562744 PMC389659

[ece38558-bib-0013] Fredericks, L. R. , Lee, M. D. , Crabtree, A. M. , Boyer, J. M. , Kizer, E. A. , Taggart, N. T. , Roslund, C. R. , Hunter, S. S. , Kennedy, C. B. , Willmore, C. G. , Tebbe, N. M. , Harris, J. S. , Brocke, S. N. , & Rowley, P. A. (2021). The species‐specific acquisition and diversification of a K1‐like family of killer toxins in budding yeasts of the Saccharomycotina. PLoS Genetics, 17, e1009341. 10.1371/journal.pgen.1009341 33539346 PMC7888664

[ece38558-bib-0014] Freese, E. B. , Chu, M. I. , & Freese, E. (1982). Initiation of yeast sporulation of partial carbon, nitrogen, or phosphate deprivation. Journal of Bacteriology, 149, 840–851. 7037742 10.1128/jb.149.3.840-851.1982 PMC216470

[ece38558-bib-0015] Gao, J. , Chau, S. , Chowdhury, F. , Zhou, T. , Hossain, S. , McQuibban, G. A. , & Meneghini, M. D. (2019). Meiotic viral attenuation through an ancestral apoptotic pathway. Proceedings of the National Academy of Sciences of the United States of America, 116, 16454–16462. 10.1073/pnas.1900751116 31266891 PMC6697816

[ece38558-bib-0016] Gao, J. , Chau, S. , & Meneghini, M. D. (2019). Viral attenuation by Endonuclease G during yeast gametogenesis: Insights into ancestral roles of programmed cell death? Microbial Cell, 7, 32–35. 10.15698/mic2020.02.705 32025511 PMC6993124

[ece38558-bib-0017] Gerke, J. , Lorenz, K. , & Cohen, B. (2009). Genetic interactions between transcription factors cause natural variation in yeast. Science, 323, 498–501. 10.1126/science.1166426 19164747 PMC4984536

[ece38558-bib-0018] Gilbert, D. G. (1980). Dispersal of yeasts and bacteria by *Drosophila* in a temperate forest. Oecologia, 46, 135–137. 10.1007/BF00346979 28310639

[ece38558-bib-0019] Herskowitz, I. , & Jensen, R. E. (1991). Putting the HO gene to work: practical uses for mating‐type switching. Methods in Enzymology, 194, 132–146. 2005783 10.1016/0076-6879(91)94011-z

[ece38558-bib-0020] Honigberg, S. M. , & Purnapatre, K. (2003). Signal pathway integration in the switch from the mitotic cell cycle to meiosis in yeast. Journal of Cell Science, 116, 2137–2147. 10.1242/jcs.00460 12730290

[ece38558-bib-0021] Horthorn, T. , Bretz, F. , & Westfall, P. (2008). Simultaneous inference in general parametric models. Biometrical Journal, 50, 346–363. 10.1002/bimj.200810425 18481363

[ece38558-bib-0022] Joy, J. B. (2013). Symbiosis catalyses niche expansion and diversification. Proceedings of the Royal Society B: Biological Sciences, 280(1756), 20122820. 10.1098/rspb.2012.2820 PMC3574373 23390106

[ece38558-bib-0023] Knight, S. J. , & Goddard, M. R. (2016). Sporulation in soil as an overwinter survival strategy in *Saccharomyces cerevisiae* . FEMS Yeast Research, 16, fov102. 26568201 10.1093/femsyr/fov102 PMC5815064

[ece38558-bib-0102] Lorenz, K. , & Cohen, B. A. (2014). Causal variation in yeast sporulation tends to reside in a pathway bottleneck. PLoS Genetics, 10, e1004634. 10.1371/journal.pgen.1004634 25211152 PMC4161353

[ece38558-bib-0024] Magwene, P. M. , Kayikci, O. , Granek, J. A. , Reininga, J. M. , Scholl, Z. , & Murray, D. (2011). Outcrossing, mitotic recombination, and life‐history trade‐offs shape genome evolution in *Saccharomyces cerevisiae* . Proceedings of the National Academy of Sciences of the United States of America, 108, 1987–1992. 21245305 10.1073/pnas.1012544108 PMC3033294

[ece38558-bib-0025] Marquina, D. , Santos, A. , & Peinado, J. M. (2002). Biology of killer yeasts. International Microbiology, 5, 65–71. 10.1007/s10123-002-0066-z 12180782

[ece38558-bib-0026] Mata, J. , Lyne, R. , Burns, G. , & Bahler, J. (2002). The transcriptional program of meiosis and sporulation in fission yeast. Nature Genetics, 32, 143–147. 10.1038/ng951 12161753

[ece38558-bib-0027] Neiman, A. M. (2005). Ascospore formation in the yeast *Saccharomyces cerevisiae* . Microbiology and Molecular Biology Reviews, 69, 565–584. 16339736 10.1128/MMBR.69.4.565-584.2005 PMC1306807

[ece38558-bib-0028] Neiman, A. M. (2011). Sporulation in the budding yeast *Saccharomyces cerevisiae* . Genetics, 189, 737–765. 22084423 10.1534/genetics.111.127126 PMC3213374

[ece38558-bib-0029] Otto, S. P. , & Lenormand, T. (2002). Resolving the paradox of sex and recombination. Nature Reviews Genetics, 3, 252–261. 10.1038/nrg761 11967550

[ece38558-bib-0030] Peter, J. , De Chiara, M. , Friedrich, A. , Yue, J.‐X. , Pflieger, D. , Bergström, A. , Sigwalt, A. , Barre, B. , Freel, K. , Llored, A. , Cruaud, C. , Labadie, K. , Aury, J.‐M. , Istace, B. , Lebrigand, K. , Barbry, P. , Engelen, S. , Lemainque, A. , Wincker, P. , … Schacherer, J. (2018). Genome evolution across 1,011 *Saccharomyces cerevisiae* isolates. Nature, 556, 339–344. 10.1038/s41586-018-0030-5 29643504 PMC6784862

[ece38558-bib-0031] Piccirillo, S. , & Honigberg, S. M. (2010). Sporulation patterning and invasive growth in wild and domesticated yeast colonies. Research in Microbiology, 161, 390–398. 10.1016/j.resmic.2010.04.001 20420901 PMC2897909

[ece38558-bib-0032] Primig, M. , Williams, R. M. , Winzeler, E. A. , Tevzadze, G. G. , Conway, A. R. , Hwang, S. Y. , Davis, R. W. , & Esposito, R. E. (2000). The core meiotic transcriptome in budding yeasts. Nature Genetics, 26, 415–423. 10.1038/82539 11101837

[ece38558-bib-0033] R Development Core Team . (2020). R: A language and environment for statistical computing. R Foundation for Statistical Computing.

[ece38558-bib-0034] Reuter, M. , Bell, G. , & Greig, D. (2007). Increased outbreeding in yeast in response to dispersal by an insect vector. Current Biology, 17, R81–R83. 10.1016/j.cub.2006.11.059 17276903

[ece38558-bib-0035] Rodriguez‐Cousino, N. , Gomez, P. , & Esteban, R. (2013). L‐A‐lus, a new variant of the L‐A totivirus found in wine yeasts with Klus killer toxin‐encoding Mlus double‐stranded RNA: Possible role of killer toxin‐encoding satellite RNAs in the evolution of their helper viruses. Applied and Environment Microbiology, 79, 4661–4674. 10.1128/AEM.00500-13 PMC3719527 23728812

[ece38558-bib-0036] Schmitt, M. J. , & Breinig, F. (2006). Yeast viral killer toxins: Lethality and self‐protection. Nature Reviews Microbiology, 4, 212–221. 10.1038/nrmicro1347 16489348

[ece38558-bib-0037] Starmer, W. T. , & Fogleman, J. C. (1986). Coadaptation of *Drosophila* and yeasts in their natural habitat. Journal of Chemical Ecology, 12, 1037–1055. 10.1007/BF01638995 24307046

[ece38558-bib-0038] Stefanini, I. , Dapporto, L. , Berna, L. , Polsinelli, M. , Turillazzi, S. , & Cavalieri, D. (2016). Social wasps are a *Saccharomyces* mating nest. Proceedings of the National Academy of Sciences of the United States of America, 113, 2247–2251. 26787874 10.1073/pnas.1516453113 PMC4776513

[ece38558-bib-0039] Sudakaran, S. , Kost, C. , & Kaltenpoth, M. (2017). Symbiont acquisition and replacement as a source of ecological innovation. Trends in Microbiology, 25, 375–390. 10.1016/j.tim.2017.02.014 28336178

[ece38558-bib-0040] Thomasson, K. M. , Franks, A. , Teotonio, H. , & Proulx, S. R. (2021). Testing the adaptive value of sporulation in budding yeast using experimental evolution. Evolution, 75, 1889–1897. 10.1111/evo.14265 34029382

[ece38558-bib-0101] Tomar, P. , Bhatia, A. , Ramdas, S. , Diao, L. , Bhanot, G. , & Sinha, H. (2013). Sporulation genes associated with sporulation efficiency in natural isolates of yeast. PLoS One, 8, e69765. 10.1371/journal.pone.0069765 23874994 PMC3714247

[ece38558-bib-0041] Wickner, R. B. (1974). “Killer character” of *Saccharomyces cerevisiae*: Curing by growth at elevated temperature. Journal of Bacteriology, 117, 1356–1357. 10.1128/jb.117.3.1356-1357.1974 4591956 PMC246622

[ece38558-bib-0042] Wickner, R. B. (1996). Double‐stranded RNA viruses of *Saccharomyces cerevisiae* . Microbiological Reviews, 60, 250–265. 10.1128/mr.60.1.250-265.1996 8852903 PMC239427

[ece38558-bib-0103] Wickner, R. B. , & Edskes, H. K. (2015). Yeast killer elements hold their hosts hostage. PLoS Genetics, 11, e1005139. 10.1371/journal.pgen.1005139 25973796 PMC4431855

[ece38558-bib-0043] Zeyl, C. , & Bell, G. (1997). The advantage of sex in evolving yeast populations. Nature, 388, 465–468. 10.1038/41312 9242403

[ece38558-bib-0044] Zhu, Y. S. , Kane, J. , Zhang, X. Y. , Zhang, M. , & Tipper, D. J. (1993). Role of the gamma component of preprotoxin in expression of the yeast K1 killer phenotype. Yeast, 9, 251–266. 8488726 10.1002/yea.320090305

